# Greatly isolated heterogeneous circulating tumor cells using hybrid engineered cell membrane-camouflaged magnetic nanoparticles

**DOI:** 10.1186/s12951-024-02514-4

**Published:** 2024-05-08

**Authors:** Xinbang Jiang, Xiangyun Zhang, Chen Guo, Zhuang Liu, Xiaofang Guo, Ziying Tian, Zimeng Wang, Jingxuan Yang, Xinglu Huang, Lailiang Ou

**Affiliations:** Key Laboratory of Bioactive Materials for the Ministry of Education, College of Life Sciences, Nankai University, Tianjin, 300071 China

**Keywords:** Cancer metastasis, Circulating tumor cells, Heterogeneity, Genetic engineering, Hybrid cell membrane

## Abstract

**Background:**

Circulating tumor cells (CTCs) are considered as a useful biomarker for early cancer diagnosis, which play a crucial role in metastatic process. Unfortunately, the tumor heterogeneity and extremely rare occurrence rate of CTCs among billions of interfering leukocytes seriously hamper the sensitivity and purity of CTCs isolation.

**Methods:**

To address these, we firstly used microfluidic chips to detect the broad-spectrum of triple target combination biomarkers in CTCs of 10 types of cancer patients, including EpCAM, EGFR and Her2. Then, we constructed hybrid engineered cell membrane-camouflaged magnetic nanoparticles (HE-CM-MNs) for efficient capture of heterogeneous CTCs with high-purity, which was enabled by inheriting the recognition ability of HE-CM for various CTCs and reducing homologous cell interaction with leukocytes. Compared with single E-CM-MNs, HE-CM-MNs showed a significant improvement in the capture efficiency for a cell mixture, with an efficiency of 90%. And the capture efficiency of HE-CM-MNs toward 12 subpopulations of tumor cells was ranged from 70 to 85%. Furthermore, by using HE-CM-MNs, we successfully isolated heterogeneous CTCs with high purity from clinical blood samples. Finally, the captured CTCs by HE-CM-MNs could be used for gene mutation analysis.

**Conclusions:**

This study demonstrated the promising potential of HE-CM-MNs for heterogeneous CTCs detection and downstream analysis.

**Graphical abstract:**

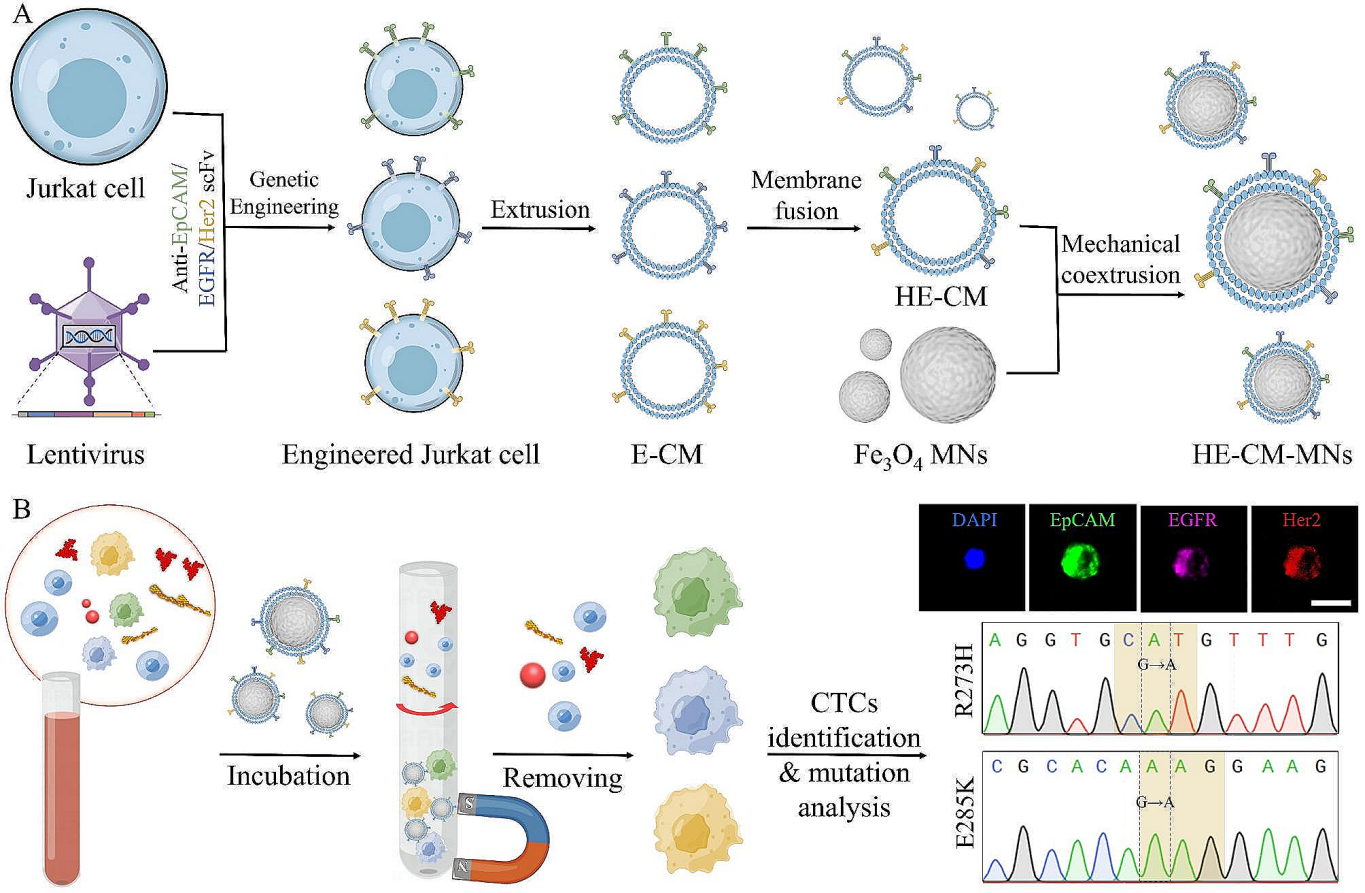

**Supplementary Information:**

The online version contains supplementary material available at 10.1186/s12951-024-02514-4.

## Introduction

Cancer is one of the leading causes of death worldwide, and cancer metastasis contributes more than 90% of cancer-related mortality [1–4]. For cancer metastasis occurrence, detached tumor cells from primary tumor serve as ‘seed’ to enter circulation system and migrate to distant ‘soil’ to form new tumor focus [5–7]. These tumor cells in blood are known as circulating tumor cells (CTCs), which are considered an important player in cancer metastasis and a liquid biopsy biomarker for early cancer diagnosis and therapy guidance [8, 9]. Hence, multiple isolation technologies have been developed to isolate CTCs from peripheral blood samples, mainly based on physical separation and biological enrichment [10, 11]. Among them, immunomagnetic nanoparticles (IMNs), which employ magnetic nanoparticles (MNs) as a separation medium and combine with targeting molecules to recognize CTCs, are one of the most frequently used platforms [12, 13]. Unfortunately, owing to the highly heterogeneous nature of tumor cells and extremely rare CTCs among with a high background of blood cells, these challenges have led to a compromised sensitivity in CTCs detection and low purity for IMNs platform, which may result in the loss of some CTCs and inconvenience for researchers in selecting a single tumor cell from numerous leukocytes for downstream analysis [5]. For example, although approved for clinical use, the CellSearch system can only recognize CTCs overexpressing epithelial cell adhesion molecule (EpCAM) and still yield low purity [14, 15]. Moreover, some studies report that CTCs may exhibit the epithelial-to-mesenchymal transition (EMT) behavior to lose their epithelial properties, leading to low or no EpCAM expression in some CTCs [16–18]. Therefore, the traditional use of EpCAM alone as a targeting ligand may unable to meet the requirements of capturing different subpopulation of CTCs. Hence, a new platform for isolation of heterogeneous CTCs with high purity is urgent need to develop, which is of great significance.

With the rapid advances in nanotechnology, especially in employment of cell membrane-derived nanovesicles and cell membrane-camouflaged nanoparticles, many promising technologies have been developed for cancer therapy and diagnosis [19]. Based on cell membrane coating, these nanomaterials inherit the properties of the source cell membrane and confer extensive performances such as extended circulation time, immune escape and reduced interaction with homologous cells [20, 21]. Some studies have demonstrated that nanomaterials coated with cell membrane derived from white blood cells (WBCs) can reduce the nonspecific binding interaction between materials and WBCs and have a good biocompatibility [22–24]. Thus, some WBCs cell membrane-camouflaged IMNs have been developed to high-performance isolation of CTCs. Generally, chemical coupling is the one of most commonly methods for conjugating targeting molecules to cell membrane [25, 26]. However, the fabrication of biomimetic IMNs by chemical conjugation may encounter obstacles of unstable molecules coupling on cell membrane and compromise the biological activity of antibodies. Furthermore, the construction of the biomimetic IMNs is costly, time-consuming and multi-processes, which may result in batch differences and impede clinical application. Hence, the development of new methods for inexpensive, simple and high-performance separation of CTCs would be of vital significance. The genetic engineering approach that expressing highly specific target protein ligands onto the cell membrane through gene editing is one of the most commonly used approaches for constructing engineered cell membrane (E-CM), which could maintain the native structure, orientation and complete biological activity of antibodies, thus enabling high-performance and specific recognition for various biomedical applications [27]. For example, some researchers have constructed a tumor nano-vaccine by expressing single-chain fragment variable (scFv) of anti-CD40 antibody on the surface of cell membrane, enhancing the targeted antitumor efficiency in tumor models [28]. Meanwhile, when the scFv expresses on the cell membrane of leukocytes, it can efficiently capture CTCs with high purity owing to the targeting ability of scFv and cell homology.

Firstly, we separated CTCs in clinical samples by size effect of microfluidic chips and found that EpCAM/EGFR/Her2 were widely presented as biomarkers in CTCs from peripheral blood of various cancer patients. Together, we previous constructed a E-CM-camouflaged platform to isolate a single subpopulation of CTCs with high-performance and one step preparation [29]. Based on the advantages, in order to detect tumor cells with heterogeneous marker expressions or subpopulations, we developed hybrid E-CM-camouflaged magnetic nanoparticles (HE-CM-MNs) with high sensitivity and purity. Firstly, cell membrane derived from genetically engineered Jurkat-anti EpCAM/ (epidermal growth factor receptor) EGFR/ (epidermal growth factor receptor-2) Her2 scFv cells were fused and then coated onto MNs to prepare HE-CM-MNs. Due to inherit the targeting properties of these scFv, HE-CM-MNs could specifically bind to heterogeneous (EpCAM^+^, EGFR^+^ or Her2^+^) CTCs. In addition, the interaction between HE-CM-MNs and WBCs could be reduced owing to their homology. After that, HE-CM-MNs were employed to isolate CTCs from clinical blood samples and the captured CTCs were successfully revealed and analyzed for TP53 gene mutations. This work confirmed the great potential of HE-CM-MNs for clinical detection of rare heterogeneous CTCs, improving the detection sensitivity in cancer diagnosis and metastasis monitoring.

## Results and discussion

### Analysis of target combination and fabrication of engineered jurkat cells

**Fig. 1:**
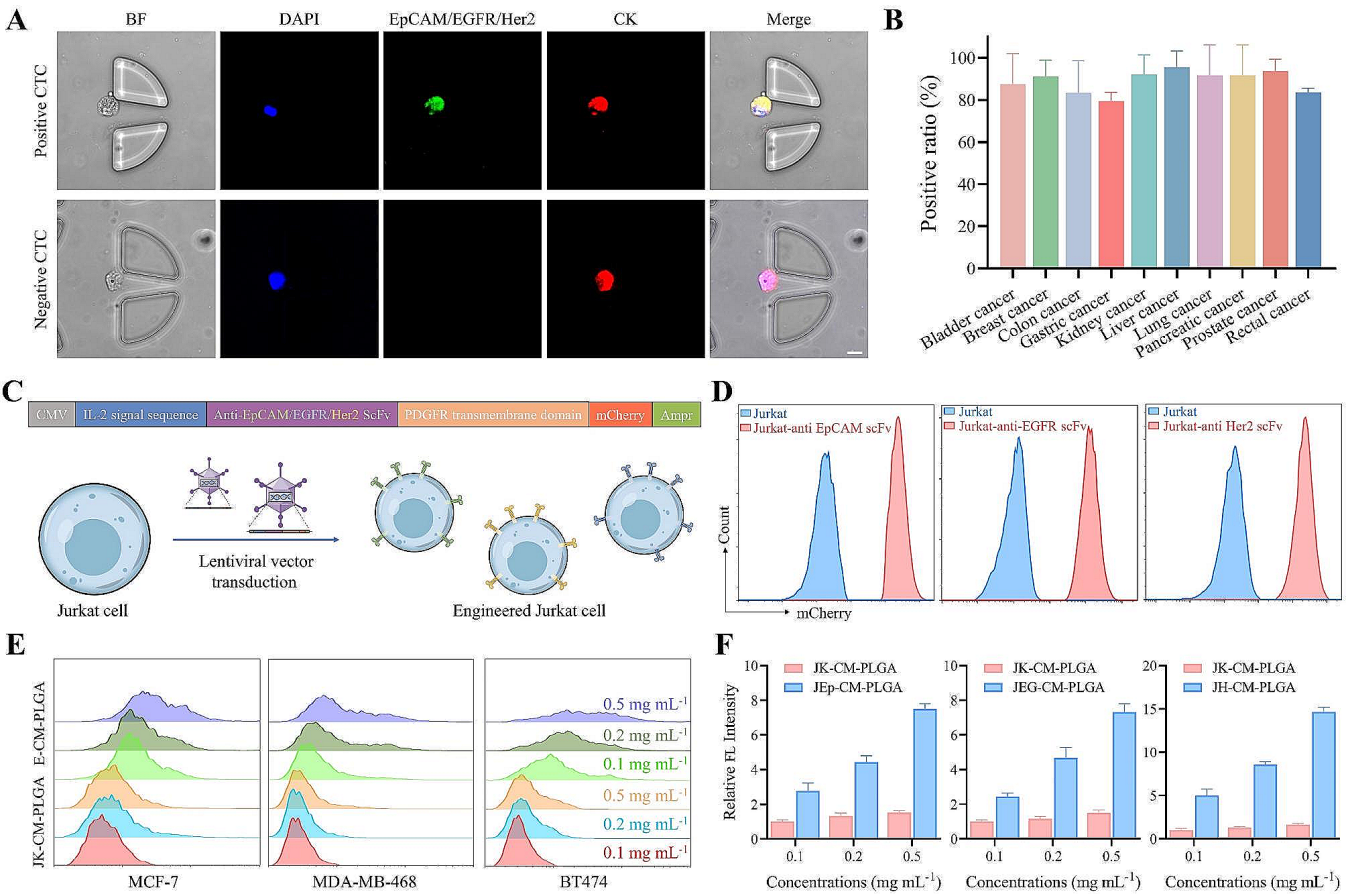
Universality of EpCAM/EGFR/Her2 combination and the preparation of engineered Jurkat cells. **(A)** Representative images of EpCAM/EGFR/Her2 positive CTC and negative CTC. Scale bar, 10 μm. **(B)** The positive rate of CTCs expressing EpCAM/EGFR/Her2 in all CTCs. Data represent as mean ± s.d. (*n* ≥ 3). **(C)** The main components of plasmid and the obtainment of various engineered Jurkat cells by corresponding lentiviral vector transduction treatment. **(D)** Flow cytometry analysis of mCherry fluorescence signal on different engineered Jurkat cells. **(E)** Flow cytometry and **(F)** quantitative analysis of the interaction between different CM-PLGA nanoparticles and cells. Data represent as mean ± s.d. (*n* ≥ 4)

In order to detect the heterogeneous CTCs, it is necessary to determine the ability of candidate surface biomarkers to meet the sensitivity requirement for CTCs detection. Most previous studies have used a combination of anti-EpCAM, anti-EGFR and anti-Her2 antibodies to high-performance detection of CTCs [30, 31]. In this study, to verify the universality of the three target molecules, CTCs were isolated from blood samples of clinical cancer patients with a microfluidic chip based on the size difference between CTCs and WBCs [31], and the protein expression level of the three molecules was analyzed using immunostaining. As shown in Fig. 1A, EpCAM/EGFR/Her2 positive CTCs were defined as having green and red fluorescence, while negative EpCAM/EGFR/Her2 CTCs solely possessing red fluorescence. Among various cancer blood samples, the positive rate of EpCAM/EGFR/Her2 CTCs in all CTCs was 70-100% (Fig. 1B), which demonstrated that the expression of the three targets was widespread in heterogeneous CTCs.

Given the advantages of EpCAM/EGFR/Her2 combination in overcoming the heterogeneity of CTCs and the homology of WBCs, we next sought to design corresponding triple-targeted molecules on leukocytes cell membrane by taking advantage of gene editing to detect heterogeneous CTCs. Firstly, to construct engineered Jurkat cells that could stably express anti EpCAM/EGFR/Her2 scFv onto the cell membrane, respectively, we used gene editing method to display three kinds of scFv on the surface of wild-type Jurkat cells. In addition to the interest scFv genes, the plasmid also included five other gene domains (Fig. 1C). Cytomegalovirus (CMV) promoter ensured the transcription of downstream genes and IL-2 signal sequence guided the targeting ligands to display natural orientation on the surface of cells. PDGFR transmembrane domain and ampicillin resistance (Ampr) gene were used to anchor scFv proteins onto the cell membrane and screened the required plasmid, respectively. mCherry protein served as a reporter for the construction of engineered cells and provided positive signal for cell sorting. After lentivirus transduction treatment, the expression of mCherry protein on engineered cells was confirmed by flow cytometry and fluorescence imaging, while hardly found in wild-type Juakat cells (Fig. 1D and Figure S1A), which indicated the successful construction of engineered Jurkat cells. To obtain the E-CM, the engineered cells were treated by a combination of hypotonic lysis, mechanical ultrasound, and high-speed centrifugation. To further confirm the binding ability, different E-CMs were coated onto poly(lactic-co-glycolic acid) (PLGA) nanoparticles and incubated with tumor cells expressing the cognate antigens for each ligand (Figure S1B). Compared with wild-type Jurakt cell membrane-camouflaged (JK-CM) PLGA nanoparticles, a bright DiI signal appeared on EpCAM (MCF-7)/EGFR (MDA-MB-468)/Her2 (BT474) expressing tumor cells after incubation with engineered Jurkat-anti EpCAM/EGFR/Her2 cell membrane-camouflaged (JEp-CM/JEG-CM/JH-CM) PLGA nanoparticles, respectively, verifying the feasibility of targeting cell recognition (Fig. 1E). Furthermore, the fluorescence intensity was increased with the concentration of E-CM-PLGA nanoparticles, while almost no change in JK-CM-PLGA nanoparticles (Fig. 1F).

### Physicochemical characterizations of E-CM-MNs

**Fig. 2:**
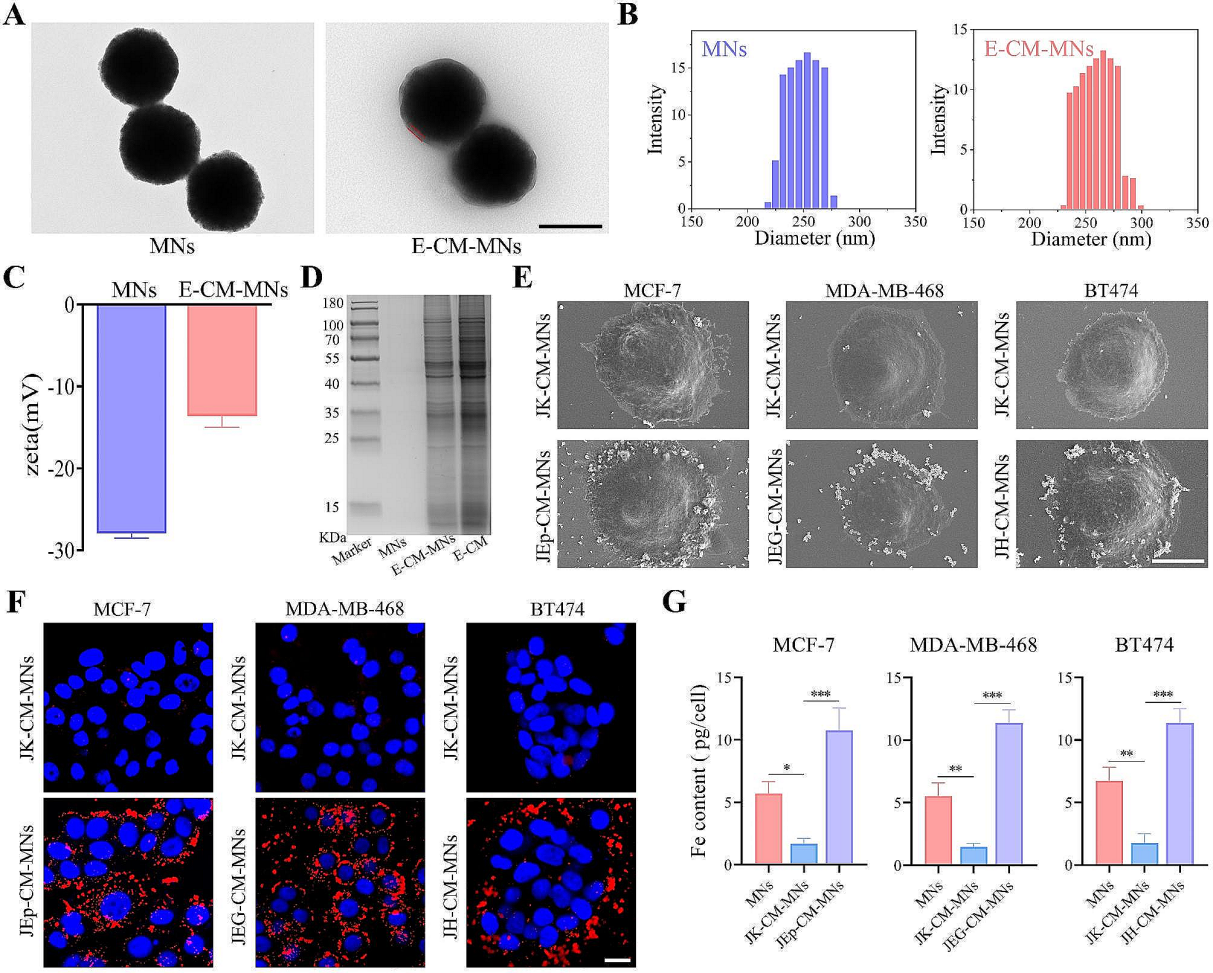
Characterization of E-CM-MNs. **(A)** TEM images of MNs and E-CM-MNs. Scale bar, 200 nm. **(B)** Size distribution and **(C)** zeta potential of MNs and E-CM-MNs. Data represent as mean ± s.d. (*n* = 4). **(D)** SDS-PAGE protein analysis of MNs, E-CM-MNs and E-CM. **(E)** SEM images of tumor cells incubated with different CM-MNs. Scale bar, 10 μm. **(F)** Fluorescence images of the interaction between various CM-MNs and different cells. Scale bar, 20 μm. **(G)** ICP-OES detection of the interaction between various nanoparticles and different cells. Data represent as mean ± s.d. (*n* = 3). *p < 0.05, **p < 0.01, ***p < 0.001

To construct E-CM-MNs, Fe_3_O_4_ MNs and E-CM were blended and coextruded using a mini extruder. As shown in Fig. 2A, the E-CM-MNs showed a core-shell structure that was characteristic of cell membrane-camouflaged MNs, while not presented on naked MNs. Moreover, the bright red signal demonstrated the successful coating of cell membrane on MNs after incubation with DiI (Figure S2A). The average size of E-CM-MNs was increased to ∼ 260 nm and the zeta potential of MNs changed from  -27.9 mV to -13.6 mV after E-CM coating (Fig. 2B C). Additionally, E-CM-MNs possessed excellent colloidal stability over course of 15 days (Figure S2B). SDS-PAGE result of the extracted proteins illustrated that the E-CM was successfully transferred to the MNs (Fig. 2D). Besides, the saturation magnetization value of E-CM-MNs was 63.2 emu g^-1^, which was equivalent to MNs (Figure S2C). The excellent magnetic strength enabled the collection of 99.3% E-CM-MNs within 1 min, providing a strong foundation for the rapid separation of E-CM-MNs in biological fluids (Figure S2D).

To verify the interaction between different E-CM-MNs and cells expressing its cognate receptor, MCF-7, MDA-MB-468 and BT474 cells were co-incubated with JEp-CM-MNs, JEG-CM-MNs and JH-CM-MNs, respectively. As shown in Fig. 2E, SEM images showed that a large amount of E-CM-MNs were observed on the cells surface expressing the corresponding receptor, with almost no visible JK-CM-MNs. The fluorescence images also demonstrated that E-CM-MNs could efficiently bind to corresponding tumor cells (Fig. 2F). In order to quantitatively analyze the interaction between different nanoparticles and various cells, we used ICP-OES to detect the Fe content. As shown in Fig. 2G, the cell membrane coating could significantly reduce the nonspecific binding between naked nanoparticles and cells, while the E-CM coating could facilitate the E-CM-MNs to target cancer cells with cognate receptor.

### Highly efficient isolation of heterogeneous CTCs using HE-CM-MNs

**Fig. 3:**
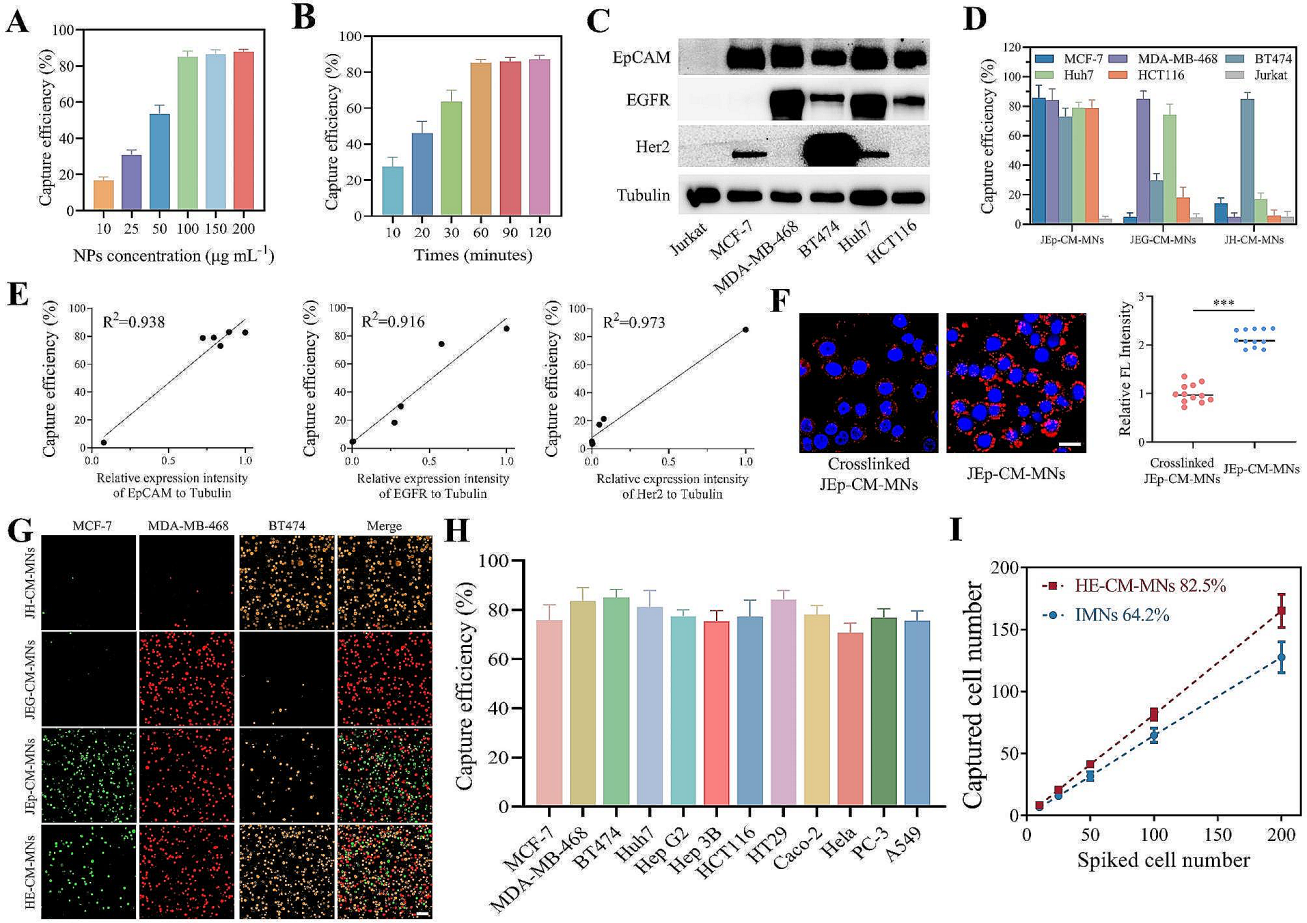
The capture capability of various E-CM-MNs toward cancer cells. **(A)** Different concentrations and **(B)** incubation times of JEp-CM-MNs for capturing MCF-7 cells. Data represent as mean ± s.d. (*n* = 4). **(C)** Representative Western blot images showing the expression of EpCAM, EGFR and Her2 on different cells. **(D)** Capture efficiency of various cells using different E-CM-MNs. Data represent as mean ± s.d. (*n* = 4). **(E)** Linear correlation analysis showing capture efficiency of various E-CM-MNs toward cells with different relative protein expression levels. **(F)** Fluorescence images and quantitative analysis of the binding interaction between tumor cells and JEp-CM-MNs treated with or without glutaraldehyde. Scale bar, 20 μm. Data represent as mean ± s.d. (*n* = 12). ***p < 0.001. **(G)** Fluorescence images showing captured cells from a mixture of MCF-7, MDA-MB-468 and BT474 cells (1:1:1) incubated with different E-CM-MNs. Scale bar, 100 μm. **(H)** The capture efficiency of HE-CM-MNs toward various tumor cells. Data represent as mean ± s.d. (*n* = 4). **(I)** Capture efficiency of IMNs or HE-CM-MNs at different quantities of tumor cells in artificial blood samples. Data represent as mean ± s.d. (*n* = 3)

Having successfully confirmed the functionality of different E-CM-MNs to target cells with corresponding receptor, we evaluated the capture efficiency of E-CM-MNs toward tumor cells. As shown in Fig. 3A, the capture efficiency increased from 16.7 to 85.1% with the concentration increased from 10 to 100 µg mL^-1^. Meanwhile, the separation could be accomplished with 60 min (Fig. 3B). After choosing suitable capture conditions, different E-CM-MNs were incubated with DiI-labelled cells with corresponding receptor and bright-field microscopy reveled that E-CM-MNs could capture targeting cells (Figure S3A). After that, we analyzed the capture stability of JEp-CM-MNs toward targeting cells, and the result showed that the capture ability of JEp-CM-MNs could be stabilized within 10 days (Figure S3B). To further evaluate the enrichment efficiency of different E-CM-MNs toward different cells, we used six kinds of cells with different levels of EpCAM, EGFR and Her2 protein expression (Fig. 3C). As shown in Fig. 3D, JEp-CM-MNs, JEG-CM-MNs and JH-CM-MNs could selectively capture EpCAM/EGFR/Her2 positive cells, respectively. Also, the linear analysis exhibited a good correlation between the capture efficiency of different E-CM-MNs toward cells with different relative antigen expression levels (Fig. 3E). In addition to the high recognition ability of scFv, the membrane fluidity may play a vital role on above excellent capture efficiency. To evaluate the effect of membrane fluidity, tumor cells were incubated with JEp-CM-MNs treated with or without glutaraldehyde. As a result, the binding affinity of crosslinked JEp-CM-MNs toward tumor cells was significantly reduced (Fig. 3F), which would compromise the enrichment performance (Figure S3C).

In addition to a single E-CM-camouflaged MNs for capturing a single subpopulation of tumor cells, HE-CM-MNs formed by a combination of three kinds of E-CM (JEp-CM, JEG-CM and JH-CM at a 1:1:1 mass ratio) were employed to test the capture efficiency in comparison to the use of JEp-CM-MNs or JEG-CM-MNs or JH-CM-MNs alone. Cell mixture of DiO-labelled MCF-7, DiI-labelled MDA-MB-468 and DiD-labelled BT474 (1:1:1) was incubated with various E-CM-MNs in capture medium. As shown in Fig. 3G, JEG-CM-MNs and JH-CM-MNs could only capture MDA-MB-468 and BT474 cells, respectively. Although JEp-CM-MNs could capture these cells owing to the high EpCAM expression, the representative fluorescent images and quantitative analysis showed that the number of captured tumor cells and the capture efficiency of JEp-CM-MNs was lower than that of HE-CM-MNs (Fig. 3G and Figure S3D). These results illustrated that a single kind of E-CM-MNs was insufficient to effectively capture CTCs with heterogeneous antigen expressions. Although some cells with low expression of EpCAM (like A549 cells) or EGFR (like Hep G2 and PC-3 cells) or Her2 (like MCF-7 and MDA-MB-468 cells) (Figure S4), the HE-CM-MNs could also maintain high capture efficiency toward these cells (Fig. 3H). This confirmed the high capture performance of HE-CM-MNs toward heterogeneous CTCs and suggested the vital significance of employing a combination of various targeting ligands for enriching tumor cells with different antigens expression.

Due to the scarcity of CTCs, we firstly spiked rare tumor cells into PBS for the capture experiment. As shown in Figure S3E, HE-CM-MNs and IMNs showed similar cell capture performance. Furthermore, to simulate the clinical blood samples, tumor cells were spiked into healthy donor blood. The result showed that HE-CM-MNs maintained the high performance to capture CTCs and exhibited higher capture efficiency than IMNs (Fig. 3I), which may be attributed to the fact that cell membrane coating could hinder the formation of protein corona thus protecting the binding affinity of targeting ligands in the blood [32, 33].

### HE-CM-MNs reduced nonspecific adsorption of protein and WBCs

**Fig. 4:**
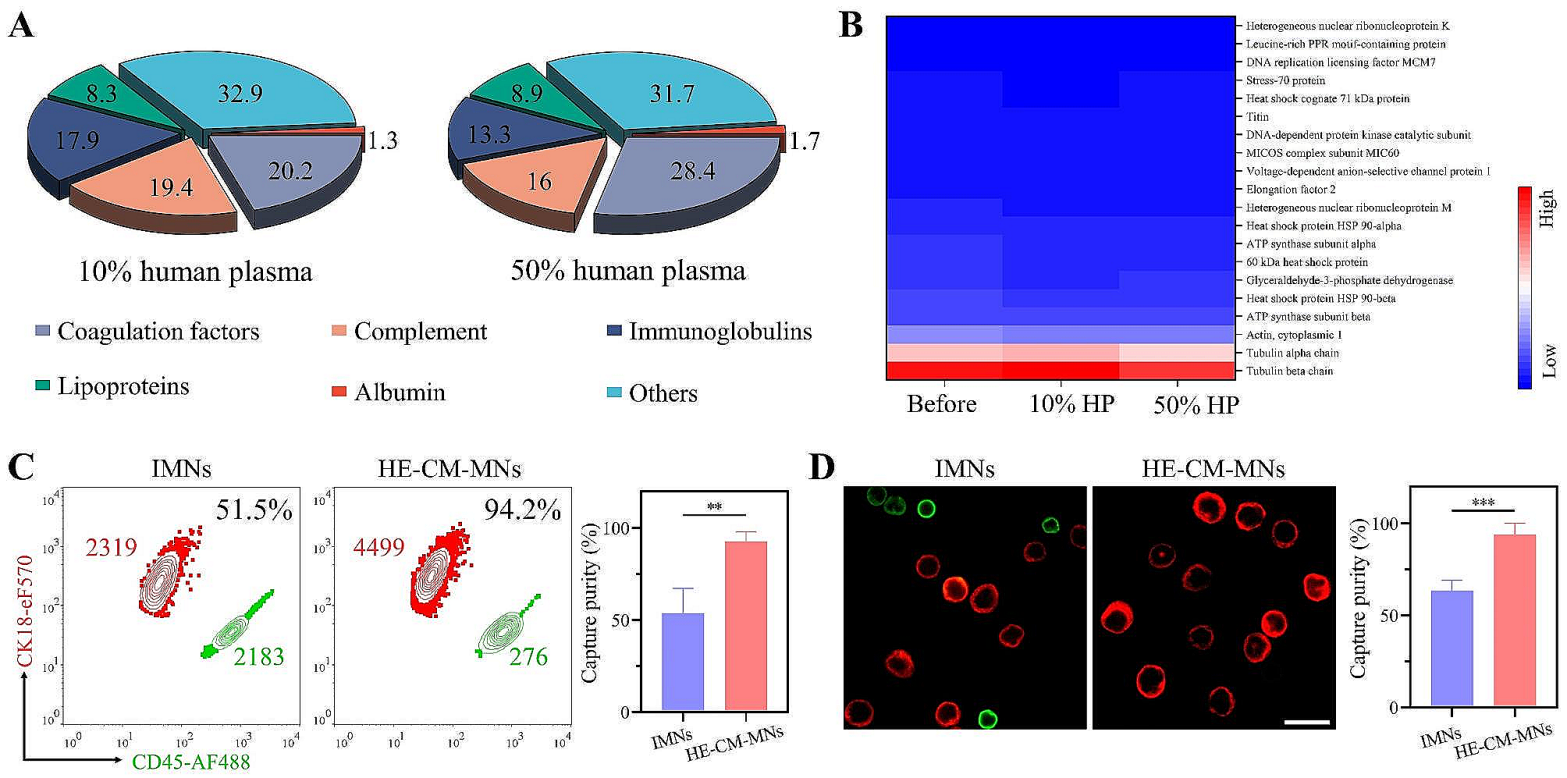
Cell membrane coating reduced the nonspecific interaction between particles and plasma protein or WBCs. **(A)** Components of the protein corona on IMNs after incubation with 10 or 50% human plasma. **(B)** Heat map of the most abundant proteins on HE-CM-MNs before and after incubation with 10 or 50% human plasma. **(C)** Flow cytometry and quantitative analysis and **(D)** fluorescence images and quantitative analysis of captured cells in artificial clinical samples. Scale bar, 10 μm. Data represent as mean ± s.d. (*n* ≥ 3). **p < 0.01. ***p < 0.001

When encountering the circulation system, the surface of nanoparticles was rapidly covered by proteins to form protein corona, which strongly influenced the biological property of antibody functionalized nanoparticles and reduced their targeting ability [34]. After confirming the E-CM coating, we hypothesized that the coating could suppress the formation of protein corona. To verify it, IMNs and HE-CM-MNs were co-incubated with 10% or 50% human plasma, respectively. Subsequently, the extracted proteins from above nanoparticles-protein complex were analyzed by LC-MS/MS. After the co-incubation with plasma protein, the component groups of proteins extracted from the IMNs-corona complex were mainly composed of coagulation factors, complement and immunoglobulins (Fig. 4A). As shown in Fig. 4B, in contrast, the components of HE-CM-MNs showed almost unchanged after plasma incubation, which illustrated that E-CM coating could effectively suppress the binding of proteins to MNs.

On the other hand, leucocytes could avoid forming cluster with cognate cells, which led to the hypothesis that the E-CM coating could restrain the binding interaction between MNs and leucocytes. To verify this, IMNs and HE-CM-MNs were incubated with a mixture of tumor cells and WBCs. The flow cytometry analysis showed that the tumor cells capture purity of commercial IMNs was 51.5%, which could improve to 94.2% using HE-CM-MNs as a capturing medium (Fig. 4C). Furthermore, in order to simulate clinical CTCs capture purity testing, the cancer cells were mixed in whole blood samples. As shown in Fig. 4D, HE-CM-MNs selectively captured tumor cells with negligible leucocytes, while the presence of leucocytes could be observed in IMNs. Also, the quantification analysis demonstrated that the capture purity of HE-CM-MNs toward tumor cells was increased to 93.9%, compared with that of 63.5% for IMNs. These results suggested that the E-CM coating could effectively reduce nonspecific adsorption of WBCs. In summary, by comparing the target molecule, identification quantity of subpopulations, capture efficiency and capture purity of various CTCs isolation techniques, we found that the HE-CM-MNs were a potential platform for capturing CTCs (Table S1).

### Isolation of CTCs in mouse models of breast cancer

**Fig. 5:**
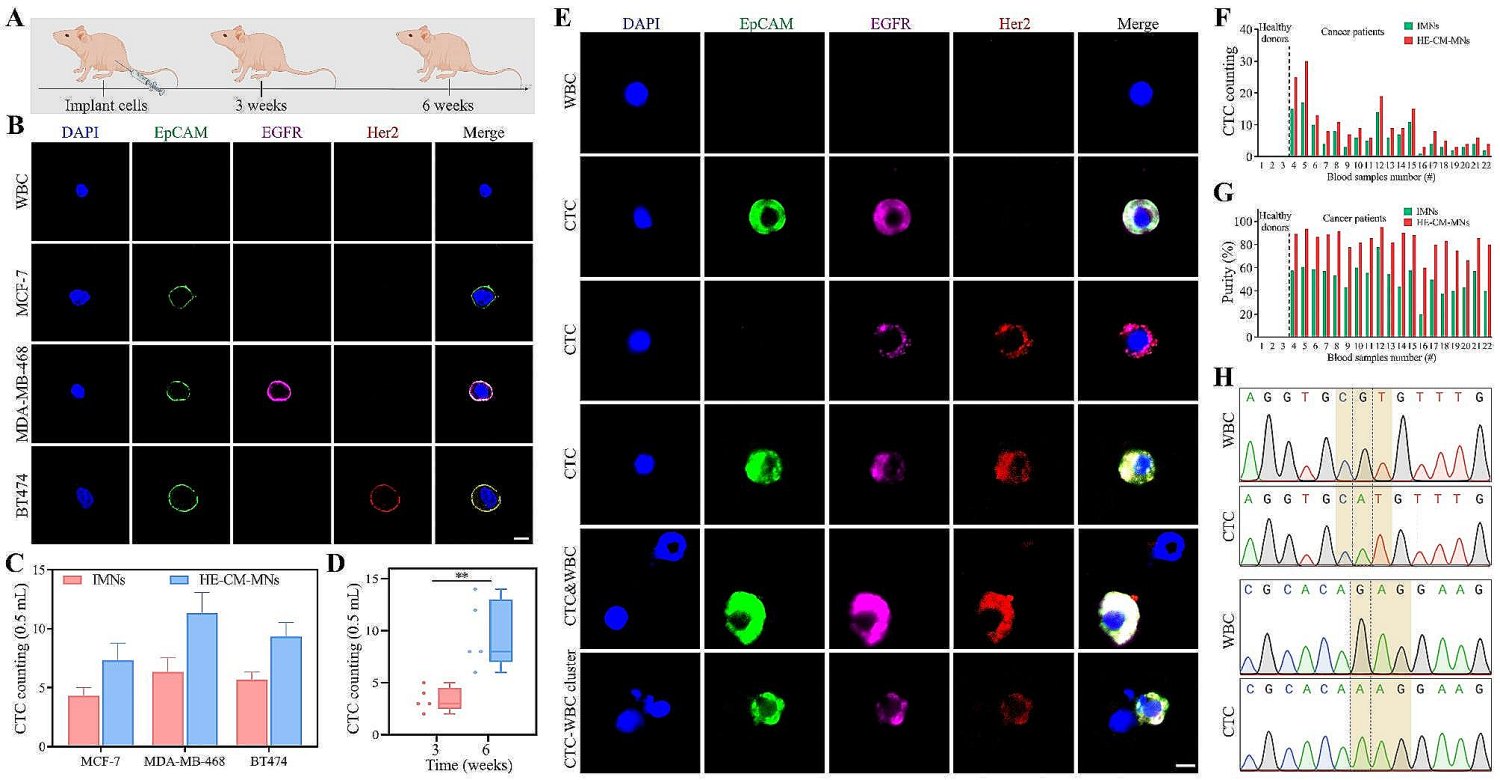
Detecting CTCs using IMNs or HE-CM-MNs. **(A)** Schematic design of mouse cancer models. **(B)** Representative images of captured CTCs and a normal mouse cell. Scale bar, 5 μm. **(C)** CTC counting in six weeks mouse cancer models. Data represent as mean ± s.d. (*n* = 3). **(D)** CTC counting using HE-CM-MNs in xenograft models at different time periods. Data represent as mean ± s.d. (*n* = 5). **p < 0.01. **(E)** Representative images of captured cells by HE-CM-MNs. Scale bar, 5 μm. **(F)** CTC counting and **(G)** capture purity in cancer peripheral blood. **(H)** Sanger sequencing results. The 818G/A (R273H) and 853G/A (E285K) point mutation were detected at the single cell level

To deeply test the practical applications of HE-CM-MNs, we next analyzed the capture performance toward blood samples obtained from mice bearing xenografted human breast cancer tumors. We used three cell lines to represent the spectrum of human breast cancer subpopulations: MCF-7, MDA-MB-468 and BT474. Mouse blood samples were collected when tumor cells were implanted in the mice within three or six weeks (Fig. 5A) and pretreated to remove red blood cells, followed by procedures of cell capture using IMNs or HE-CM-MNs. For fluorescence detection, the captured cells were immune-stained with a combination of antibodies against EpCAM, EGFR and Her2, and cell nuclei were stained with DAPI. The characterizations of WBCs and CTCs were DAPI^+^/EpCAM^−^/EGFR^−^/Her2^−^ and DAPI^+^/EpCAM^+^ or EGFR^+^ or Her2^+^, respectively (Fig. 5B). Due to the co-expression of EpCAM in these cancer cells, the count of captured CTCs was calculated based on the number of cells expressing EpCAM. The result showed that the number of captured CTCs per 500 µL of blood was ranged from 3 to 14 and HE-CM-MNs had a better capture performance toward tumor cells, compared with IMNs (Fig. 5C). Furthermore, the number of cancer cells was increased with time of cell implantation (Fig. 5D).

### Detection of CTCs in clinical testing

To evaluate the clinical application, blood samples from healthy volunteers and cancer patients were pretreated to remove red blood cells and incubated with commercial IMNs and our HE-CM-MNs (Table S2). Then, the captured cells were immune-stained with anti-EpCAM, anti-Her2, anti-EGFR antibodies and DAPI and the number of CTCs and WBCs isolated from clinical samples was counted (Fig. 5E). In 19 out of 19 cancer patient blood samples, HE-CM-MNs exhibited higher capture performance and higher capture purity than IMNs, while no CTCs were observed in healthy donors (Fig. 5F and G).

Before the downstream analysis, we tested the biocompatibility of HE-CM-MNs toward tumor cells. After incubation with different concentration of HE-CM-MNs, the proliferation ability of cells remained almost unchanged (Figure S5A). Moreover, the captured cells were stained with Calcein-AM/PI and the result showed a low number of dead cells (Figure S5B). Furthermore, the captured cells were detached and cultured in complete medium to evaluate the cell proliferation capacity. As shown in Figure S5C, the re-cultured cells maintained a similar proliferative ability as the untreated cells. After confirming the good biocompatibility of HE-CM-MNs, the captured WBCs and CTCs were recovered and analyzed for TP53 gene mutation. As shown in Fig. 5H, the DNA sequencing of CTCs confirmed that the metastatic cancer cells were positive for the 818G/A (R273H) and 853G/A (E285K) point mutation. In contrast, WBCs captured from patients exhibited no TP53 gene mutation. Therefore, these results showed that the high-performance isolation of CTCs by HE-CM-MNs could enable the downstream gene mutations analysis.

## Conclusion

In summary, a new platform for simple, sensitive and high efficiency with high-purity isolation of heterogeneous cancer cells was successfully constructed by taking advantage of the genetic engineering technology and cell homology. Three different types of scFv (anti-EpCAM/EGFR/Her2) were displayed on cell membrane and as a shell coated onto MNs to form HE-CM-MNs. The biomimetic HE-CM shell significantly reduced the nonspecific binding of protein and WBCs, maintaining the targeting ability of HE-CM-MNs toward heterogeneous tumor cells. Furthermore, HE-CM-MNs could specifically and sensitively capture CTCs from clinical blood samples of cancer patients. More importantly, the captured CTCs could be recovered and analyzed for downstream cellular analysis such as TP53 gene mutations. This study confirmed that the HE-CM-camouflaged nanoparticles-based technology showed promising prospects for the design of cell membrane-based materials, offering an innovative platform to benefit early cancer diagnosis, metastasis monitoring and personalized therapy.

## Experimental methods

### Analysis the rate of positive CTCs with EpCAM/EGFR/Her2 expression

A volume of 1 mL clinical cancer patient blood sample was collected by EDTA K2 tubes and pretreated to remove red blood cells. The pellet was re-suspended in 1mL PBS and loaded into the single-cell trapping chip at a flow rate of 0.5 mL h^-1^. The trapped cells were treated with 4% paraformaldehyde (PFA) for 10 min and permeabilized with 0.1% PBS-Triton for 10 min. Then the cells were incubated overnight at 4℃ with AF488-conjugated anti-EpCAM/EGFR/Her2 and eFluor 570-conjugated anti-cytokeratin antibodies. After washing with PBS, nuclear DNA was labeled with DAPI and detected by fluorescence microscope.

### Anti-EpCAM/EGFR/Her2 scFv expressing Jurkat cell line

Different lentivirus with anti-EpCAM/EGFR/Her2 scFv expressing plasmids were constructed to express scFv protein on the surface of Jurkat cells. The variable gene from the light domain and heavy domain was conjugated by (G_4_S)_3_ and linked with PDGFR transmembrane domain. mCherry protein was evaluated for scFv expressing cells. Next, three kinds of plasmids were transfected into the HEK 293T cells along with CMV and VSVG packaging plasmids, respectively. After 16 h of incubation, the supernatant medium was replaced with complete medium and collected at 48 and 72 h. After mixing, the collected medium was centrifuged to remove impurity. Then, the collected medium and culture medium (4:1) were co-cultured with cells for 24 h. Finally, the scFv expressing cells were sorted, recovered and re-cultured.

### The binding interaction between various E-CM-camouflaged PLGA nanoparticles and tumor cells expressing corresponding receptor

Firstly, PLGA nanoparticles were constructed with slight modification as previous study [35]. Then, the PLGA nanoparticles were mixed with cell membrane, extracted with a Mini-Extruder Set and the obtained nanoparticles were labelled with DiI. After incubating with corresponding tumor cells, the mixture was centrifugated and analyzed the DiI signal binding onto cells by flow cytometry.

### **Preparation and characterization of E-CM-MNs**

To obtain E-CM-MNs, E-CM was mixed with MNs and extruded with a Mini-Extruder Set. The morphology of MNs/E-CM-MNs was characterized by TEM with negative staining using 1% uranyl acetate. Hydrodynamic diameter and zeta potential were determined by using Nano Zeta-Sizer. SDS-PAGE was employed to analyze protein components.

### The binding interaction between different MNs and tumor cells

Tumor cells were seeded onto glass slide and cultured for 24 h. After incubation with different CM-MNs for 2 h, the cells were fixed with 2% PFA and gradient dehydration by a serial of ethanol solutions (50%, 70%, 90%, and 100%). Then, the cells were frozen overnight at -80℃ and dehydrated under vacuum freezing. After complete dry, the cells were spattered with gold and observed by SEM. For fluorescence analysis, CM-MNs were labelled with DiI and incubated with cells for 2 h, and then stained with DAPI. After washing with PBS, the binding interaction was analyzed by fluorescence microscope.

### Quantitative analysis of the binding interaction between different MNs and tumor cells

Tumor cells were cultured in plate for 12 h and incubated with different MNs. Subsequently, the cells were washed with PBS, and treated with 1% Tween 80. Then, nitric acid was added and let stand for 4 h. Finally, the solution was heated at 80℃ to remove the acids and suspended in H_2_O to measure the Fe content by using an ICP-OES.

### Optimization of cell capture in PBS

Approximately 10^5^ tumor cells were resuspended in 1 mL solution and incubated with various concentrations of JEp-CM-MNs for 1 h. After magnetic separation, the uncaptured cells in supernatant were collected and counted. The number of captured cells were calculated by subtracting the number of cells remaining in the supernatant from the total number of added cells and the capture efficiency was defined as the ratio of captured cell amount against the initial cell amount. Together, to evaluate the impact of incubation time on capture efficiency, tumor cells were incubated with JEp-CM-MNs with different incubation time and then performed the capture experiment as described above.

### Capture performance evaluation of various E-CM-MNs

Approximately 10^5^ cells were suspended in 1 mL PBS and incubated with different E-CM-MNs. Then, the capture experiment and capture efficiency were performed as described above. Furthermore, the capture efficiency of various E-CM-MNs toward cells with different relative protein expression levels was performed linear correlation analysis. To get crosslinked JEp-CM-MNs, JEp-CM-MNs were fixed with 2% PFA for 30 min. Moreover, MCF-7, MDA-MB-468 and BT474 cells were pre-stained with DiO, DiI and DiD, respectively and incubated with various E-CM-MNs. After magnetic separation and washing with PBS, the captured cells were analyzed by fluorescence microscope. Finally, to evaluate the capture efficiency of HE-CM-MNs toward heterogeneous cancer cells, twelve kinds of tumor cells were incubated with HE-CM-MNs and performed the capture experiment as described above.

### Rare cell capture efficiency in artificial samples

To prepare commercial IMNs, three kinds of biotinylated anti-human EpCAM/EGFR/Her2 antibody with 5 µg mL^-1^ were incubated with 500 µg of streptavidin-conjugated MNs at 4℃ for 6 h. Then, 10–200 DiI pre-stained tumor cells were resuspended in 1 mL PBS and incubated with commercial IMNs and HE-CM-MNs as described above. After washing with PBS, the captured tumor cells were counted using fluorescence microscope and the cell capture efficiency was calculated as described above. To mimic the clinical blood samples, 10–200 DiI pre-stained tumor cells were resuspended in 1 mL of whole blood samples from healthy donors and incubated with 100 µg of IMNs or HE-CE-MNs for 1 h. Subsequently, the captured cells were counted and cell capture efficiency was calculated as described above.

### Characterization of the interaction between nanoparticles and protein

IMNs and HE-CM-MNs were individually incubated with 1 mL of 10% or 50% human plasma for 1 h. After washing with PBS, the protein-nanoparticles complex was treated with RIPA lysis buffer to obtain protein components and analyzed by liquid chromatography-mass spectrometry/mass spectrometry as previous study [36].

### Capture specificity in lysis blood samples

Approximately 1 × 10^4^ tumor cells were spiked into 1 mL of lysis blood sample and incubated with IMNs or HE-CE-MNs for 1 h. After that, the captured cells were treated with trypsinization and the obtained single cell solution were stained with AF488 anti-CD45 and eF570 anti-CK18. After washing with PBS, the cells were analyzed by flow cytometry. The capture purity was calculated by captured tumor cells against total captured cells.

### Capture specificity in artificial blood samples

Approximately 5 × 10^3^ tumor cells were spiked into 1 mL of healthy blood samples and incubated with IMNs or HE-CE-MNs for 1 h. After that, the captured cells were stained with AF488 anti-CD45 and eF570 anti-CK18 and detected by fluorescence microscope. The capture purity was calculated as described above.

### Mouse models of breast cancer

Breast cancer cells were collected, centrifuged and washed with PBS. Female balb/c nude mice (6–8 weeks) were anesthetized and their mammary fat pads were injected subcutaneously with 5 × 10^6^ tumor cells in 100 µl of 50% growth factor-reduced Matrigel using 0.3-ml insulin syringes. After specified time, blood for CTC detection was drawn via cardiac puncture, pretreated to remove red blood cells and performed the capture experiment as described above. After washing with PBS, the cells were fixed with 4% PFA for 10 min and then immunostained with AF488 anti-EpCAM, AF555 anti-Her2, AF647 anti-EGFR for 2 h. Finally, the cell nuclei were stained with DAPI for fluorescence detection.

###  CTC detection from clinical blood samples

Firstly, commercial IMNs and HE-CM-MNs were added into pretreated peripheral blood obtained from healthy volunteers or cancer patients and co-incubated for 1 h. Then, the captured cells were fixed with 4% PFA for 10 min and then immunostained with AF488 anti-EpCAM, AF555 anti-Her2, AF647 anti-EGFR for 2 h. Finally, the cell nuclei were stained with DAPI for fluorescence detection.

### Gene analysis of clinical blood sample

The cell-containing solution in the confocal dish was withdrawn by a 20 µL micropipette under fluorescence microscope. Then, the entire genome of captured single cell was amplified followed the procedure of WGA kit. TP53 gene was amplified by real-time polymerase chain reaction with primers (Forward primer: TCTCTGGCTTTGGGACCTCT, Reverse primer: GCCCCAATTGCAGGTAAAACA). Finally, the amplified DNA was analyzed by Sanger sequencing.

### Electronic supplementary material

Below is the link to the electronic supplementary material.

Supplementary Material 1

## Data Availability

No datasets were generated or analysed during the current study.
