# Supplementary material for: Greatly isolated heterogeneous circulating tumor cells using hybrid engineered cell membrane-camouflaged magnetic nanoparticles
Source: J Nanobiotechnology. 2024 May 8;22:231. doi: 10.1186/s12951-024-02514-4 (PMC11077811; doi:10.1186/s12951-024-02514-4)
Supplement: Supplementary file 1 — Supplementary Material 1 [file 12951_2024_2514_MOESM1_ESM.docx]

Supporting Information

**Greatly Isolated Heterogeneous Circulating Tumor Cells using Hybrid Engineered Cell Membrane-camouflaged Magnetic Nanoparticles**

Xinbang Jiang^1, #^, Xiangyun Zhang^1, #^, Chen Guo^1^, Zhuang Liu^1^, Xiaofang Guo^1^, Ziying Tian^1^, Zimeng Wang^1^, Jingxuan Yang^1^, Xinglu Huang^1,*^, Lailiang Ou^1,*^

^1^Key Laboratory of Bioactive Materials for the Ministry of Education, College of Life Sciences, Nankai University, Tianjin 300071, China.

^#^These authors contributed equally to this work.

^*^Corresponding to: huangxinglu@nankai.edu.cn, [ouyll@nankai.edu.cn](mailto:ouyll@nankai.edu.cn).

**Experimental Section**

Materials and Reagents

The anti EpCAM/EGFR/Her2 scFv genes were constructed by Sangon Biotech. Fugene@6 transfection reagent was purchased from Promega. Anti-human EpCAM/EGFR/Her2 and anti-human CD45 antibodies were purchased from Abcam. Dulbecco's Modified Eagle Medium (DMEM), Roswell Park Memorial Institute (RPMI) 1640 medium (RPMI 1640), 0.25% trypsin-EDTA, fetal bovine serum (FBS) and eFluor 570-conjugated anti-human Cytokeratin (eF570 anti-CK) were obtained from Thermo-Fisher. The magnetic nanoparticles were obtained from Xueyanhui Scientific Research Platform (China). Commercial streptavidin-conjugated magnetic nanobeads were purchased from BEAVER (China). Anti α-tubulin monoclonal antibody and the other reagents used were obtained from Beijing Solarbio Science & Technology Co., Ltd (China).

Cell culture

HEK 293T and A549 cells were obtained from ATCC. Jurkat, MCF-7, MDA-MB-468, BT474, HuH7, Hep G2, Hep 3B, HCT116, HT29, Caco-2, Hela and PC-3 cells were purchased from Procell Life Science & Technology Co., Ltd. Jurkat, engineered Jurkat cells and BT474 were cultured in RPMI 1640 and other cells cultured in DEME medium, supplemented with 10% FBS and 1% penicillin/streptomycin at 37℃ in 5% CO_2_ incubators.

Extraction of cell membrane

After centrifugation of scFv expressing Jurkat cells, the cells were washed and disrupted at 4℃ overnight with hypotonic buffer. The cell membrane fragment was processed with an ultrasonic cell disruption device until the suspension was clarified. After that, the suspension was centrifuged at 3,200 g for 5 minutes, and 16,000 g for 30 minutes. Finally, the precipitation was washed with PBS, dissolved in PBS and stored at -80℃.

Cell viability analysis

To test the biocompatibility, different concentrations of MNs were incubated with cells for 24 hours. The untreated cells were as a control. Then the cell viability was evaluated by CCK-8 kit. Also, the viability of captured cells was determined by calcein acetoxymethyl ester/propidium iodide and analyzed by fluorescence microscope.

Cell release and re-culture

In order to release the tumor cells, the captured cells were resuspended in trypsin and incubated at 37℃ for 2 min. After that, the released single cell solution was seeded in a 96-well plate and cultured for proliferation studies. CCK-8 was used to evaluate cell proliferation at 1, 2 and 3 days as described above.

**Animal procedures and patient samples**

All animal procedures and patient samples were performed in accordance with the guidelines in the “the interim rules for Ethical Review of Biomedical Research Involving Humans Subject” developed by the National Health Commission of the People’s Republic of China and approved by the ethics committee at Nankai University (Tianjin, China). Informed consent was obtained from human participants of this study.

Statistical analysis

All data are shown as means ± standard deviation (s.d.). The statistical difference between two groups was analyzed using Student’s t-test. One-way analysis of variance (ANOVA) followed by multiple comparisons testing was used to compare among multiple groups. All statistical analysis was two-sided, and p values less than 0.05 were considered statistically significant. The GraphPad Prism version 8.0 (GraphPad Software) software was used for the statistical calculations.


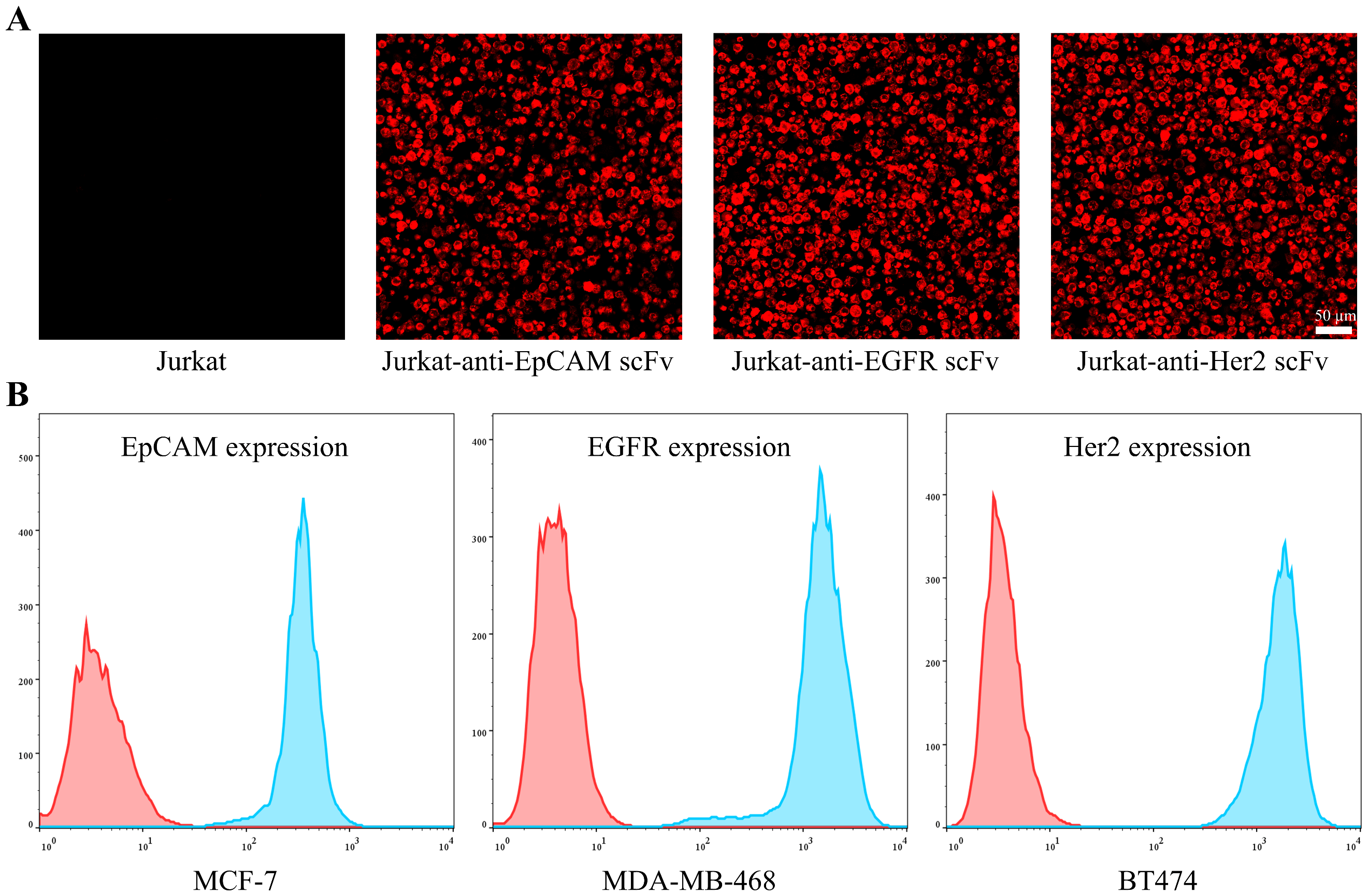


**Figure S1.** A) Fluorescence images of the mCherry signal on different Jurkat cells. B) Flow cytometry analysis of different antigen expression on various cells.


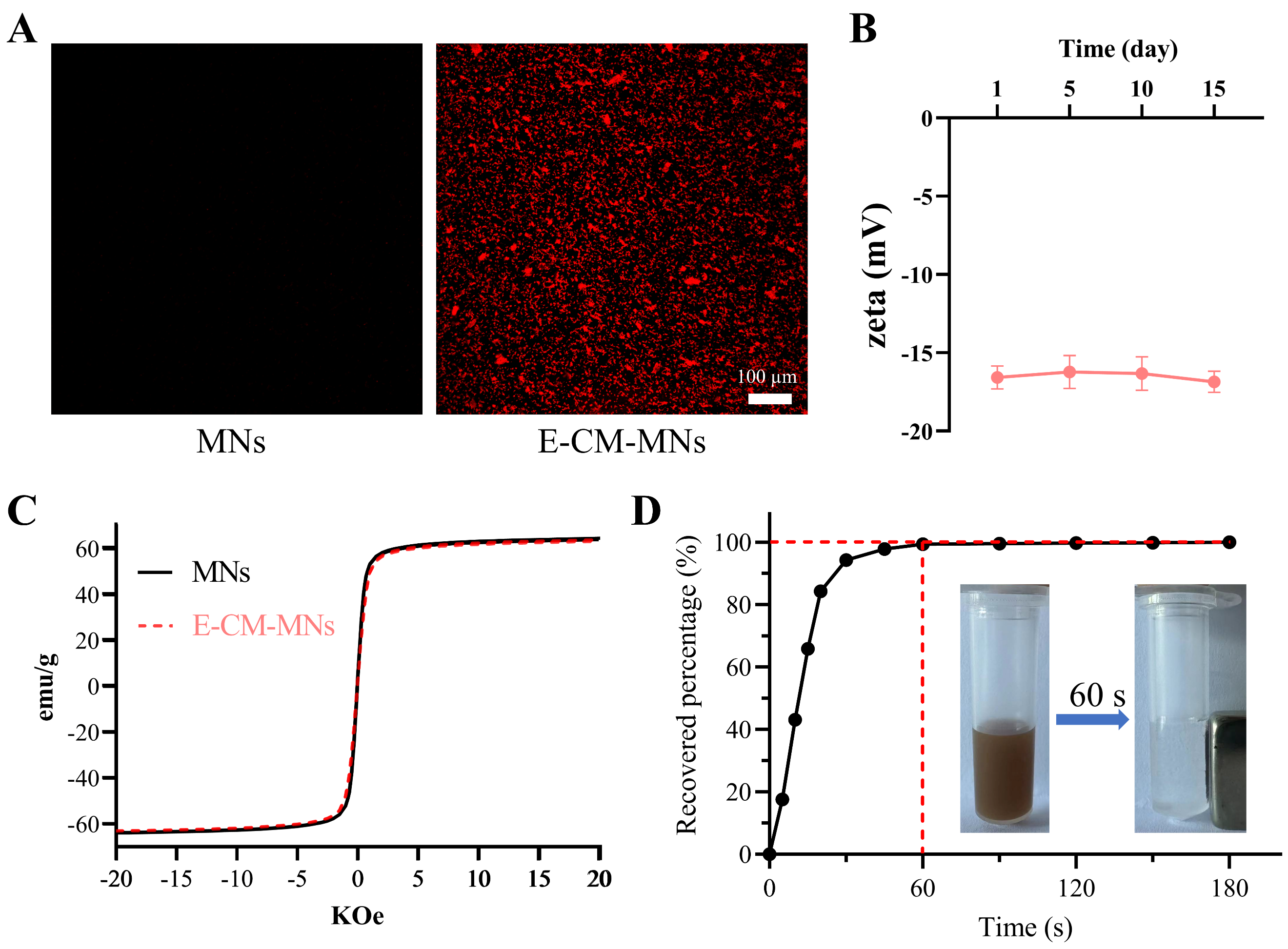


**Figure S2.** A) Fluorescence images of MNs and E-CM-MNs modified with DiI. B) Zeta potential changes of E-CM-MNs in PBS within 15 days. C) Magnetization curves of the MNs before and after cell membrane coating. D) Recovered percentage of E-CM-MNs at different attraction times with a commercial magnetic scaffold.


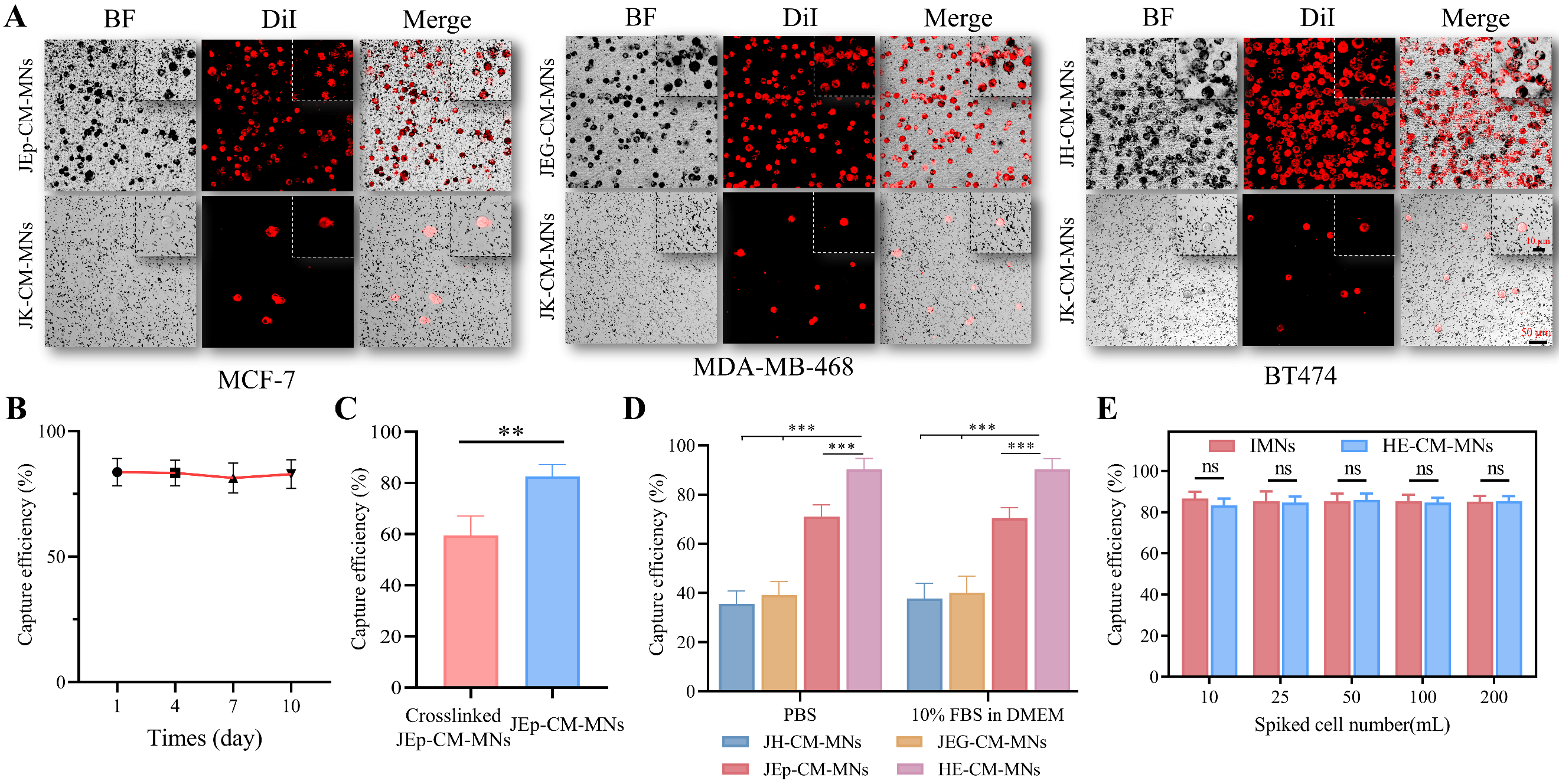


**Figure S3.** A) Fluorescence microscopy images of DiI-labelled cells binding to various CM-MNs. B) The cell capture efficiency of JEp-CM-MNs stored in PBS within 10 days. C) The capture efficiency of JEp-CM-MNs before and after crosslinking. ***p* < 0.01. D) Quantitative data on the capture efficiency of different E-CM-MNs for capturing a cell mixture population in PBS or cell culture medium. ****p* < 0.001. E) The capture efficiency of IMNs and HE-CM-MNs toward rare MDA-MB-468 cells in PBS.


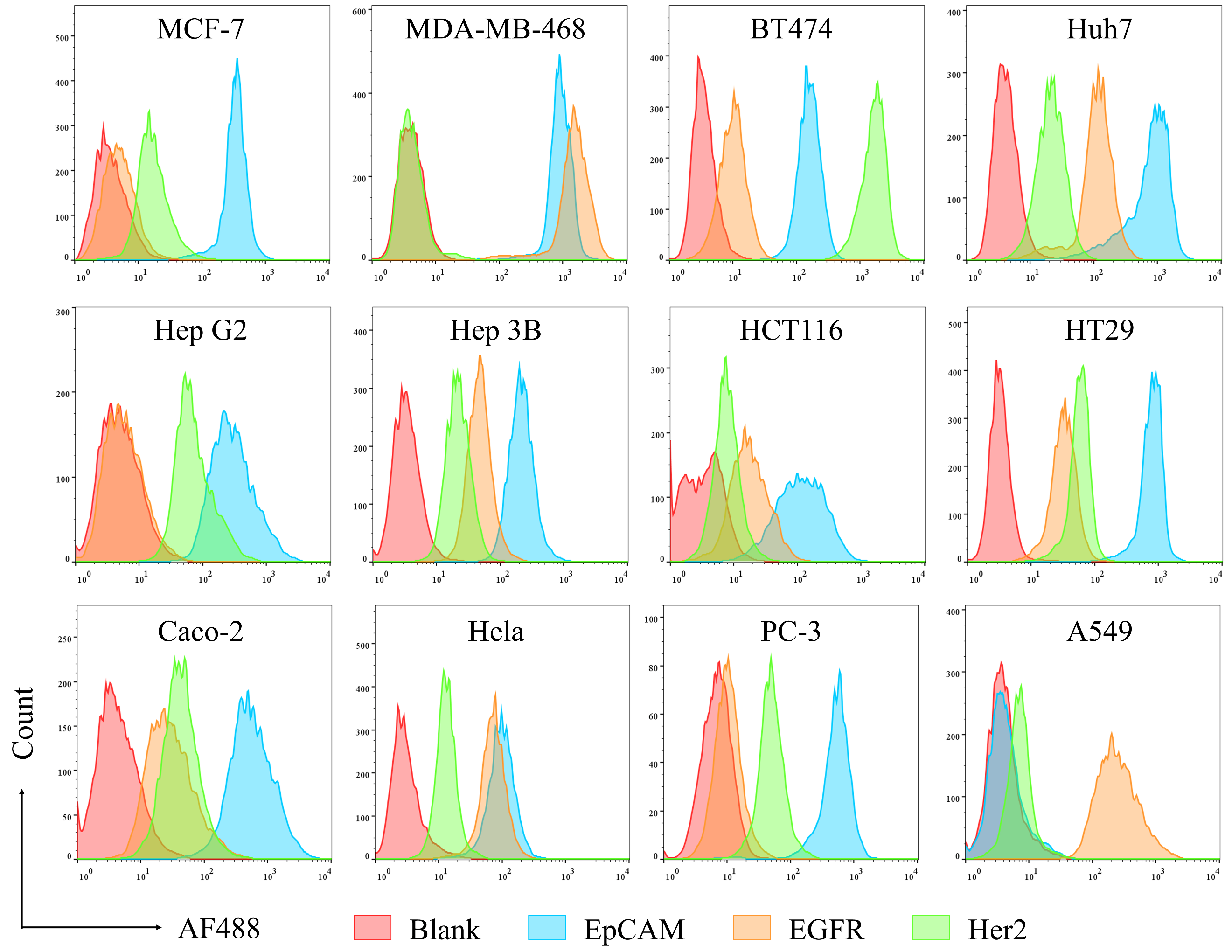


**Figure S4.** Expression of EpCAM, EGFR and Her2 on various tumor cells.


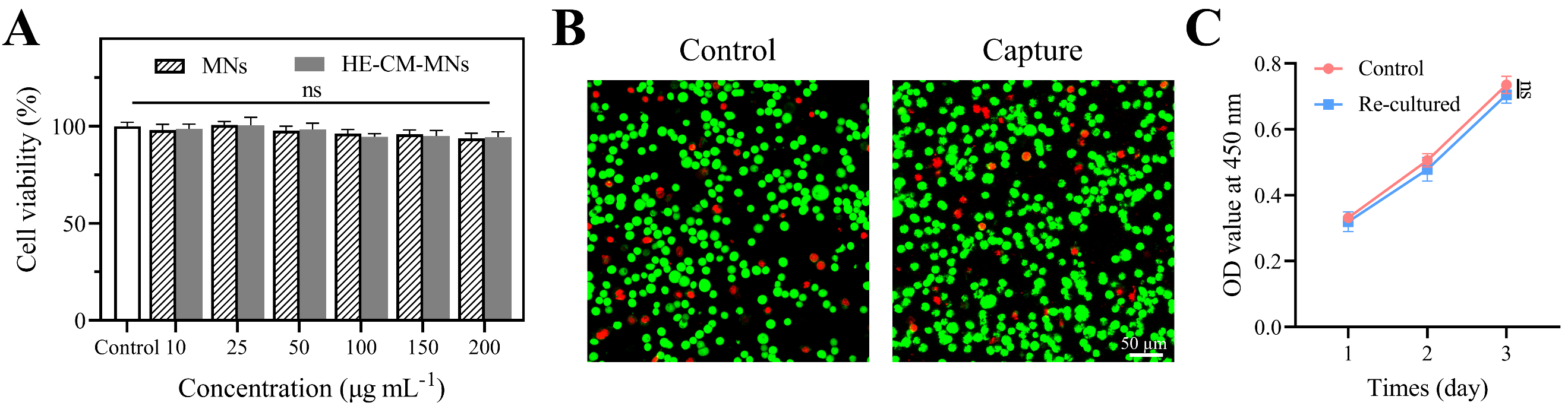


**Figure S5.** A) Cell viability assay of MDA-MB-468 cells after incubation with different concentrations of MNs or HE-CM-MNs for 24 hours. B) CLSM images of control and the captured MDA-MB-468 cells stained with calcein AM (green) and PI (red). C) The control and released cell proliferation analysis by CCK-8 assay.

**Table S1.** Various CTCs isolation techniques.

| CTCs isolation techniques | Target molecule | Identification quantity of subpopulations | Capture efficiency | Capture purity |
| --- | --- | --- | --- | --- |
| CellSearch system | Antibody | 1 | 40% | 0.5% |
| CM-Fe_3_O_4_@Au-Ab^[1]^ | Antibody | 1 | 79% | 85% |
| HM-IMBs^[2]^ | Antibody | 1 | 91.77% | 96.98% |
| IMSs^[3]^ | Antibody | 1 | 90% | - |
| F-MNPs^[4]^ | Antibody | 2 | 88.5% | - |
| BFMNPs^[5]^ | Antibody | 3 | 74.9-87.5% | 72.1-85.9% |
| FA-MNPs^[6]^ | FA | 1 | 80% | - |
| CMMS-FA-RGD^[7]^ | FA-RGD | 2 | 91% | 89% |
| CM-LM-MBs^[8]^ | Cancer CM | 1 | 75% | 92.24% |
| JE-CM-MNs^[9]^ | E-CM | 1 | 80.4% | 91.2% |
| HE-CM-MNs | E-CM | 3 | 82.5% | 94.2% |

**Table S2.** CTC counting of 1.0 mL whole blood samples collected from healthy donors or cancer patients. The purity of CTC was defined as the ratio of captured CTCs against the total number of captured cells.

| Blood sample | Healthy/  cancer | CTC counting | | CTC putity | |
| --- | --- | --- | --- | --- | --- |
|  |  | IMNs | HE-CM-MNs | IMNs | HE-CM-MNs |
| 1 | Healthy | 0 | 0 | / | / |
| 2 | Healthy | 0 | 0 | / | / |
| 3 | Healthy | 0 | 0 | / | / |
| 4 | Breast cancer | 15 | 25 | 57.69% | 89.29% |
| 5 | Breast cancer | 17 | 30 | 60.71% | 93.75% |
| 6 | Breast cancer | 10 | 13 | 58.82% | 86.67% |
| 7 | Breast cancer | 4 | 8 | 57.14% | 88.89% |
| 8 | Breast cancer | 8 | 11 | 53.33% | 91.67% |
| 9 | Breast cancer | 3 | 7 | 42.86% | 77.78% |
| 10 | Colon cancer | 6 | 9 | 60% | 81.82% |
| 11 | Colon cancer | 5 | 6 | 55.56% | 85.71% |
| 12 | Colon cancer | 14 | 19 | 77.78% | 95% |
| 13 | Lung cancer | 6 | 9 | 54.55% | 81.82% |
| 14 | Lung cancer | 7 | 9 | 43.75% | 90% |
| 15 | Lung cancer | 11 | 15 | 57.89% | 88.24% |
| 16 | Liver cancer | 1 | 3 | 20% | 60% |
| 17 | Liver cancer | 4 | 8 | 50% | 80% |
| 18 | Liver cancer | 3 | 5 | 37.5% | 83.33% |
| 19 | Liver cancer | 2 | 3 | 40% | 75% |
| 20 | Liver cancer | 3 | 4 | 42.86% | 66.67% |
| 21 | Liver cancer | 4 | 6 | 57.14% | 85.71% |
| 22 | Liver cancer | 2 | 4 | 40% | 80% |

**References**

1. Chang ZM, Zhou H, Yang C, Zhang R, You Q, Yan R, Li L, Ge M, Tang Y, Dong WF, Wang Z: **Biomimetic immunomagnetic gold hybrid nanoparticles coupled with inductively coupled plasma mass spectrometry for the detection of circulating tumor cells.** *J Mater Chem B* 2020, **8:**5019-5025.

2. Rao L, Meng QF, Huang Q, Wang Z, Yu GT, Li A, Ma W, Zhang N, Guo SS, Zhao XZ: **Platelet-Leukocyte Hybrid Membrane‐Coated Immunomagnetic Beads for Highly Efficient and Highly Specific Isolation of Circulating Tumor Cells.** *Adv Funct Mater* 2018, **28:**1803531.

3. Xiong K, Wei W, Jin Y, Wang S, Zhao D, Wang S, Gao X, Qiao C, Yue H, Ma G, Xie H: **Biomimetic Immuno-Magnetosomes for High-Performance Enrichment of Circulating Tumor Cells.** *Adv Mater* 2016, **28:**7929-7935.

4. Wang Z, Sun N, Liu H, Chen C, Ding P, Yue X, Zou H, Xing C, Pei R: **High-Efficiency Isolation and Rapid Identification of Heterogeneous Circulating Tumor Cells (CTCs) Using Dual-Antibody-Modified Fluorescent-Magnetic Nanoparticles.** *ACS Appl Mater Interfaces* 2019, **11:**39586-39593.

5. Liao Z, Han L, Li Q, Li L, Liu Y, Song Y, Tan W, Song E: **Gradient Magnetic Separation and Fluorescent Imaging‐Based Heterogeneous Circulating Tumor Cell Subpopulations Assay with Biomimetic Multifunctional Nanoprobes.** *Adv Funct Mater*, **31:**2009937.

6. Nie L, Li F, Huang X, Aguilar ZP, Wang YA, Xiong Y, Fu F, Xu H: **Folic Acid Targeting for Efficient Isolation and Detection of Ovarian Cancer CTCs from Human Whole Blood Based on Two-Step Binding Strategy.** *ACS Appl Mater Interfaces* 2018, **10:**14055-14062.

7. Li T, Li N, Ma Y, Bai YJ, Xing CM, Gong YK: **A blood cell repelling and tumor cell capturing surface for high-purity enrichment of circulating tumor cells.** *J Mater Chem B* 2019, **7:**6087-6098.

8. Chang ZM, Zhang R, Yang C, Shao D, Tang Y, Dong WF, Wang Z: **Cancer-leukocyte hybrid membrane-cloaked magnetic beads for the ultrasensitive isolation, purification, and non-destructive release of circulating tumor cells.** *Nanoscale* 2020, **12:**19121-19128.

9. Jiang X, Zhang X, Guo C, Ma B, Liu Z, Du Y, Wang B, Li N, Huang X, Ou L: **Genetically Engineered Cell Membrane‐Coated Magnetic Nanoparticles for High‐Performance Isolation of Circulating Tumor Cells.** *Adv Funct Mater* 2023, **34:**2304426.
